# P-1352. Clinical Outcomes of Cefiderocol for Treatment of Bloodstream Infections Caused by Gram-Negative Bacteria: Subgroup Analysis of the PROVE Study

**DOI:** 10.1093/ofid/ofaf695.1540

**Published:** 2026-01-11

**Authors:** Emily N Drwiega, David W Wareham, Massimo Antonelli, Stefano Verardi, Karan Gill, Anne Santerre Henriksen, Sean T Nguyen

**Affiliations:** University of Illinois Chicago, Chicago, Illinois; Queen Mary University London, London, England, United Kingdom; Università Cattolica del Sacro Cuore, Rome, Lazio, Italy; Shionogi, B.V., London, England, United Kingdom; Shionogi, London, England, United Kingdom; Shionogi BV, London, UK, London, England, United Kingdom; Shionogi Inc., Florham Park, NJ

## Abstract

**Background:**

Bloodstream infections (BSIs) are associated with increased morbidity and mortality. In this subgroup analysis of the PROVE study, the clinical outcomes of hospitalized patients with BSIs caused by Gram-negative bacteria, who were treated with cefiderocol, were evaluated.

**Figure:**
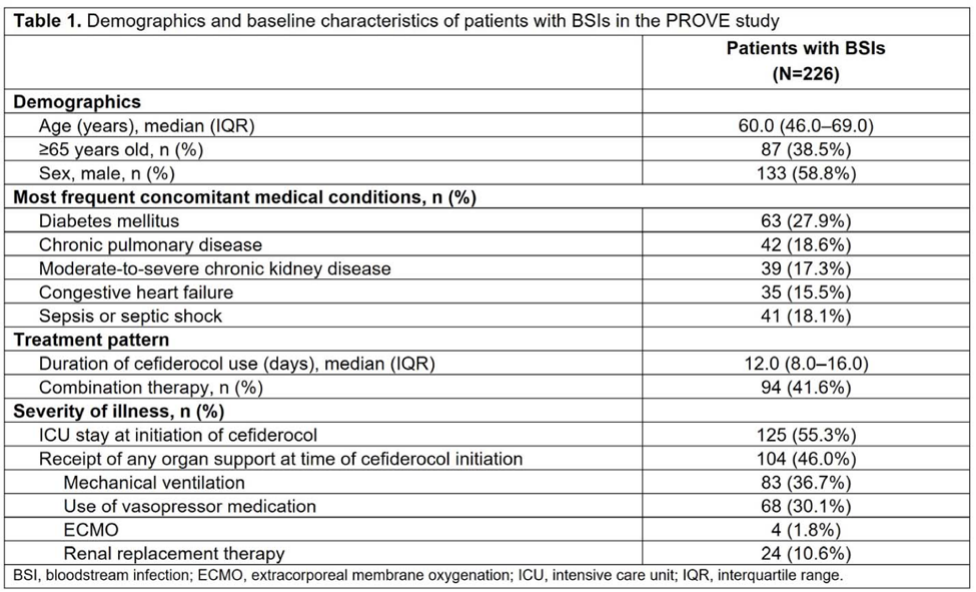

**Figure:**
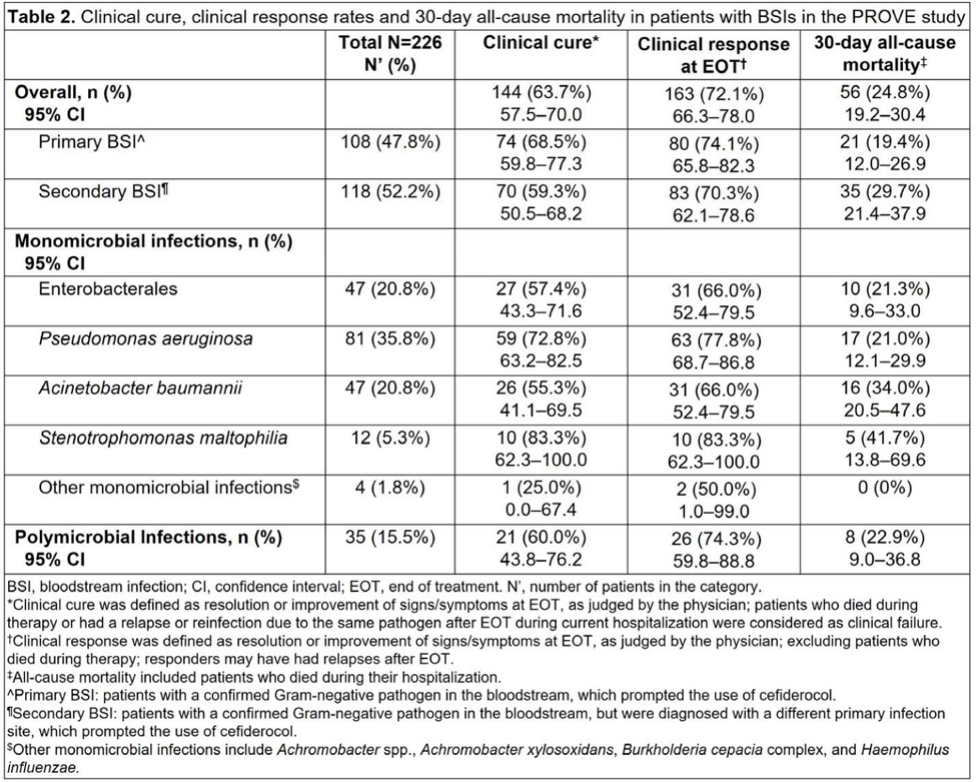

**Methods:**

In the international, observational PROVE chart review study, hospitalized patients with confirmed Gram-negative bacterial infections were treated with cefiderocol in routine practice for the first time for ≥72 hours (November 2020–July 2024). In this analysis, data of patients with primary or secondary BSIs were analyzed. Baseline demographics, clinical characteristics, cefiderocol use, clinical cure, clinical response, and all-cause mortality (ACM) rates were evaluated.

**Results:**

BSI was reported in 226 patients (primary: 47.8%; secondary: 52.2%). The median (interquartile range [IQR]) age was 60 (46–69) years and 58.8% were male (Table 1). Diabetes mellitus was the most common concomitant condition (27.9%), followed by chronic pulmonary disease (18.6%) and sepsis or septic shock (18.1%). The median (IQR) duration of cefiderocol treatment was 12 (8.0–16.0) days. At cefiderocol initiation, 55.3% of patients were in the intensive care unit and 46.0% were receiving organ support. Overall, the clinical cure rate was 63.7% (primary BSIs: 68.5%; secondary BSIs: 59.3%) and 30-day ACM was 24.8% (primary BSIs: 19.4%; secondary BSIs: 29.7%) (Table 2). Of 81 patients with monomicrobial *Pseudomonas aeruginosa* BSI, 72.8% had clinical cure. The clinical cure rates in patients with monomicrobial BSIs caused by Enterobacterales species, *Acinetobacter baumannii*, and *Stenotrophomonas maltophilia* were 57.4%, 55.3%, and 83.3%, respectively. Polymicrobial BSIs were reported in 35 patients, with clinical cure and 30-day ACM rates of 60.0% and 22.9%, respectively.

**Conclusion:**

In this large cohort of cefiderocol-treated patients, secondary BSIs were associated with more frequent treatment failure compared with primary BSIs, suggesting that patients with secondary BSIs may require more aggressive antibiotic treatment.

**Disclosures:**

David W. Wareham, MD, MRCP, FRCPath, Antimicrobial Research UK: Grant/Research Support|Rosetrees UK: Grant/Research Support Massimo Antonelli, MD, Fisher & Paykel Healthcare: Grant/Research Support|GE Healthcare: Grant/Research Support|Menarini: Advisor/Consultant|Pfizer: Advisor/Consultant|Shionogi BV: Advisor/Consultant Stefano Verardi, MD, Shionogi BV: Employee Karan Gill, Master of Science, Shionogi BV: Employee Anne Santerre Henriksen, PHD, Shionogi BV: Advisor/Consultant Sean T. Nguyen, PharmD, Shionogi Inc: Employee

